# High-throughput chemical screening to discover new modulators of microRNA expression in living cells by using graphene-based biosensor

**DOI:** 10.1038/s41598-018-29633-x

**Published:** 2018-07-30

**Authors:** Soo-Ryoon Ryoo, Yeajee Yim, Young-Kwan Kim, Il-Soo Park, Hee-Kyung Na, Jieon Lee, Hongje Jang, Cheolhee Won, Sungwoo Hong, Sung-Yon Kim, Noo Li Jeon, Joon Myong Song, Dal-Hee Min

**Affiliations:** 1 Department of Chemistry, Seoul National University, Seoul, 08826 Republic of Korea; 2 Center for RNA Research, Institute for Basic Sciences (IBS), Seoul National University, Seoul, 08826 Republic of Korea; 3 Carbon Composite Materials Research Center, Korea Institute of Science and Technology (KIST), Jeonbuk, 55324 Republic of Korea; 4 Center for Nano-Bio Measurement, Korea Research Institute of Standards and Science (KRISS), Daejeon, 34113 Republic of Korea; 5 Predictive Toxicology Department, Korea Institute of Toxicology (KIT), Daejeon, 34114 Republic of Korea; 6 Department of Chemistry, Kwangwoon University, 20 Gwangwoon-ro, Nowon-gu, Seoul 01897 Republic of Korea; 7 Institute of Biotherapeutics Convergence Technology, Lemonex Inc, Seoul, 08826 Republic of Korea; 8 Department of Chemistry, Korea Advanced Institute of Science and Technology (KAIST), Daejeon, 34141 Republic of Korea; 9 Center for Catalytic Hydrocarbon Functionalizations, Institute for Basic Science (IBS), Daejeon, 34141 Republic of Korea; 10 Department of Mechanical and Aerospace Engineering, Seoul National University, Seoul, 08826 South Korea; 11 College of Pharmacy, Seoul National University, Seoul, 08826 Republic of Korea

## Abstract

MicroRNAs (miRNAs) are important regulatory RNAs that control gene expression in various biological processes. Therefore, control over the disease-related miRNA expression is important both for basic research and for a new class of therapeutic modality to treat serious diseases such as cancer. Here, we present a high-throughput screening strategy to identify small molecules that modulate miRNA expression in living cells. The screen enables simultaneous monitoring of the phenotypic cellular changes associated with the miRNA expression by measuring quantitative fluorescent signals corresponding to target miRNA level in living cells based on a novel biosensor composed of peptide nucleic acid and nano-sized graphene oxide. In this study, the biosensor based cellular screening of 967 compounds (including FDA-approved drugs, enzyme inhibitors, agonists, and antagonists) in cells identified four different classes of small molecules consisting of (i) 70 compounds that suppress both miRNA-21 (miR-21) expression and cell proliferation, (ii) 65 compounds that enhance miR-21 expression and reduce cell proliferation, (iii) 2 compounds that suppress miR-21 expression and increase cell proliferation, and (iv) 21 compounds that enhance both miR-21 expression and cell proliferation. We further investigated the hit compounds to correlate cell morphology changes and cell migration ability with decreased expression of miR-21.

## Introduction

MicroRNAs (miRNAs) are endogenously expressed, small non-coding RNAs that regulate gene expressions at post-transcriptional level^1^. The miRNA expression is dynamically coordinated in various ways through post-transcriptional maturation processes during biogenesis and epigenetic control^2–4^. As a number of previous studies revealed that miRNA expression patterns are closely associated with cancer, viral infection and inflammatory disease^5^, miRNA is considered as an important therapeutic target in disease treatment and thus, many pharmaceutical companies are currently developing drugs targeting miRNAs to combat serious diseases such as cancer and hepatitis C^6^.

To date, strategies for therapeutic targeting of miRNAs are mainly classified into three approaches—vector to express mRNAs possessing multiple miRNA-binding sites, antisense oligonucleotide (ASO) to specifically inhibit target miRNA function (anti-miR) and small molecules to manipulate miRNA expression and/or function^7^. Among them, small molecule-based approach could hold immediate impact in drug development because if a robust screening method is available to select specific molecules which regulate target miRNA expression, one can discover new potent small molecules from chemical library or may easily relocate already FDA-approved small molecule drugs without any concerns related to ASO or vector-based approaches such as off-target effect, gene delivery system issues, and undesirable immune responses. Therefore, the discovery of new small molecules regulating target miRNA is one of the important research areas even though small molecule-based approaches bear drawbacks such as difficulty in identifying direct targets.

For the discovery of new miRNA modulators, the appropriate miRNA sensing system is required that is (1) applicable in living cells, (2) quantitative with minimized false signals, (3) capable to incorporate internal control, and (4) compatible with the high-throughput assay. Conventional strategies for miRNA sensing in cells basically rely on reporter-based miRNA assay systems in which different reporter plasmid construct should be prepared and stably transfected into cells for each distinct miRNA target, resulting in laborious preparation and time-consuming process. To overcome the challenges, our group previously developed a fluorescent miRNA sensor based on peptide nucleic acid (***P***N***A***) and nano-sized graphene oxide (***NGO***) (referred to as ‘PANGO’) in which fluorescent dye-labeled PNA probe gives a sequence-specific fluorescent signal in the presence of target miRNA inside living cells. In the PNA-NGO complex, NGO serves as a fluorescence quencher and an intracellular delivery vehicle of PNA by interacting with nucleobases of PNA through pi-pi stacking interaction and hydrogen bonding^8^.

Here, we adopted and expanded the PANGO strategy for high-throughput screening to identify small molecules that modulate miR-21 expression in living cells. miR-21 is one of the most well-known oncogenic miRNAs of which expression level is elevated in various cancers including breast, ovarian and lung cancer, and has been major target miRNA in the development of anti-miR therapeutics^5^. To discover new miR-21 modulators, we employed several advantages of the PANGO system—(i) multiplexed sensing capability that allowed addition of internal control probe recognizing housekeeping gene, glyceraldehyde 3-phosphate dehydrogenase (GAPDH) in this study, in addition to miR-21 probe, (ii) ability to give quantitative fluorescent signal corresponding to miR-21 expression level in real time while cells were alive and (iii) compatibility with high-throughput screening. During the chemical screening, changes in cell morphology and number of cells could be also easily monitored because miR-21 responsive fluorescent images of cells were obtained while cells were alive by using a high-throughput cell imaging instrument. The present study identified new small molecules that induced down- or up-modulation of miR-21 miRNA expression and further characterized hit compounds to reveal their downstream effect on cell functions and morphological changes.

## Results

### Design of PANGO miR-21 sensor for cell-based screening

**Figure 1:**
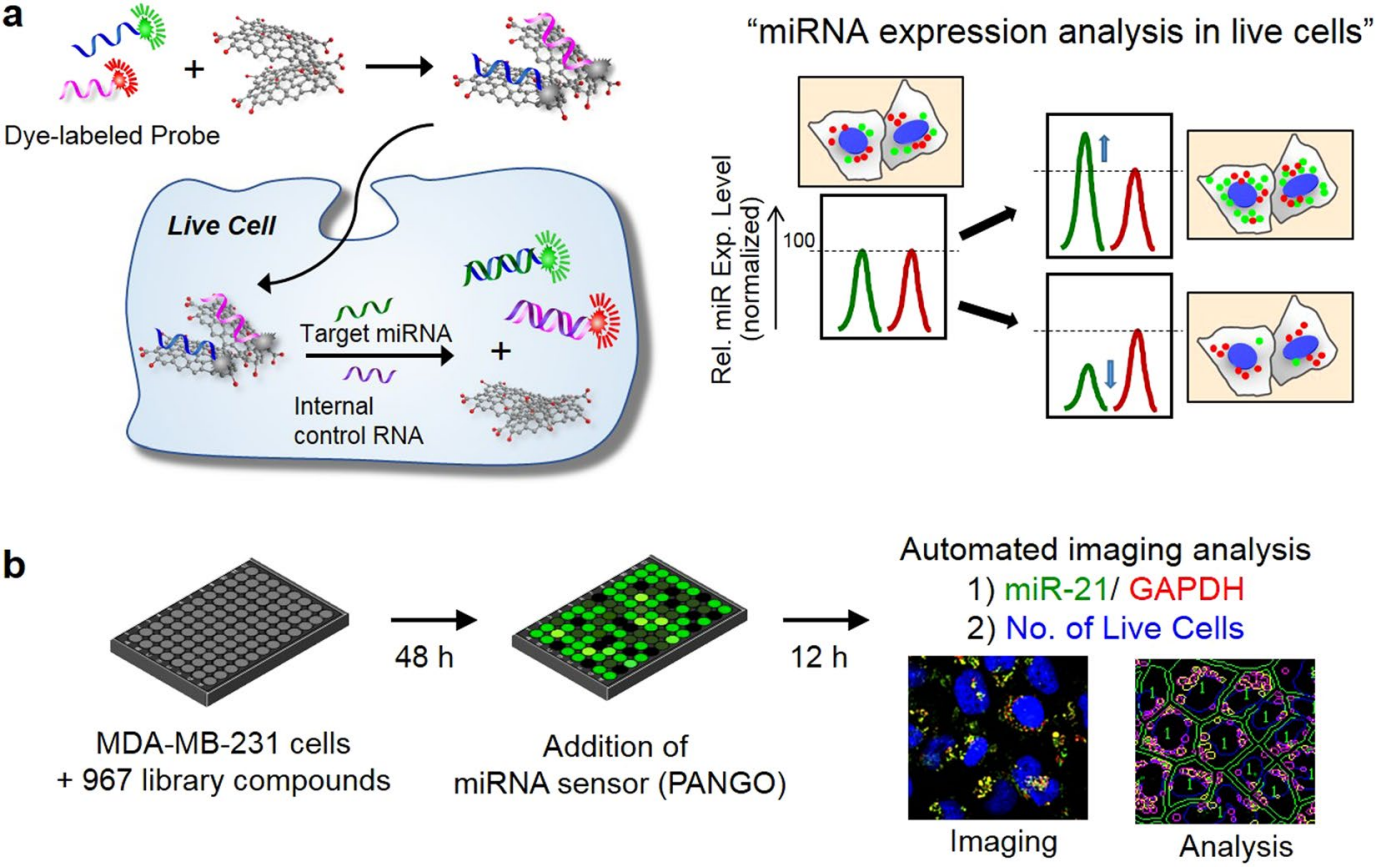
The strategy of the image based high-throughput screen for the discovery of miRNA expression regulators. (**a**) The principle of quantitative miRNA measurement in living cells. The quenched fluorescence of the probes achieved by GO binding gets recovered once the GO-PNA probe complex is internalized into the cells expressing target miRNA due to hybridization of PNA with target miRNA and simultaneous release of the PNA probe from GO. Housekeeping gene such as GAPDH serves as an internal control for precise measurement of changes in relative expression level of miRNA in living cells. (**b**) The miRNA regulator screening was performed in a 96-well plate format. Compounds from small molecule library were treated to the cells grown in 96-well plates at a final concentration of 5 mM. After 2 days of incubation, the miRNA sensor (target miRNA: miR-21) was added to each well and incubated for 12 h. The fluorescence signal gets recovered from the GO surface and the intensity gives information on the expression level of target miRNA in the cells. The relative expression level of target miRNA versus GAPDH expression and the number of live cells were analyzed from the fluorescence images.

To observe two fluorescent signals simultaneously, we prepared NGO^9^ (Supplementary Fig. S1) and two kinds of PNA probes for detection of target miRNA and GAPDH mRNA, respectively labelled with Cy3 (Cyanine 3, ex/em = 550/570 nm) and Cy5 (Cyanine 5, ex/em = 650/670 nm) (Supplementary Fig. S2a). Upon addition of the mixture of PNA probes and NGO to cells expressing target RNAs, the adsorbed probes for miR-21 and GAPDH are detached from NGO surface, showing the recovery of the quenched fluorescence signal, respectively (Fig. 1a). To evaluate the robustness of the GAPDH mRNA expression sensing probe as an internal normalization control, we next tested the PNA probe for GAPDH mRNA in living cells. We prepared the mixed solution of NGO complex containing both Cy3-PNA-21 and Cy5-PNA-GDH probes and directly added to the culture media of MDA-MB-231 cells. We observed the relatively consistent level of miR-21 expression when normalized by GAPDH signal in three sets of MDA-MB-231 cells (Supplementary Fig. S2b and c). We next determined the Z′-factor, a parameter popularly used to estimate the available signal window for an assay in terms of signal separation between positive and negative controls weighted by the errors associated with each control, to evaluate the suitability of the present PANGO sensor for the high-throughput screen. We performed the PANGO experiments with 25 positive controls (using probes for miR-21 and GAPDH) and 25 negative control (using a probe with a scrambled sequence, scPNA) in live MDA-MB-231 cells and calculated the Z′-factor using each mean and standard deviation values (Supplementary Fig. S2d). The Z′-factors of the present PANGO sensor for miR-21 and GAPDH were 0.78 and 0.77, respectively, which are considered in a suitable range for reliable high throughput screening. Generally, the Z′-factor larger than 0.75 is considered as a high-performance assay suitable for high throughput screening.

### Chemical screening to discover miR-21 modulator from small molecule library

We chose miR-21, one of the anti-apoptotic factor, as a target miRNA and MDA-MB-231 (invasive human breast cancer cell line, late-stage cancer model) as an *in vitro* model cell line to screen small molecule modulators of miR-21 expression due to its intermediate level of miR-21 among various breast cancer cell lines^10^. Oncogenic miR-21 is an anti-apoptotic factor in tumor progression and its aberrant up-regulation is closely associated with tumor formation by down-regulating tumor suppressor genes^11^. It is known that the enforced overexpression of miR-21 induced the increased cell viability and inversely, down-regulation of miR-21 by anti-miR-21 inhibited cell growth and survival^12,13^. In addition, several reports suggest that miR-21 is deeply involved in drug resistance process through the modulation of apoptosis and cancer survival signaling pathways.

In the present study, we quantitatively measured changes in miR-21 expression level and the number of cells per well at the same time after the treatment of chemical library to the cells, to evaluate cell proliferation rate as a phenotypic change of the cells under the conditions where miR-21 expression level can be altered (Fig. 1b). Chemical screening to discover miRNA expression modulators was performed in a 96-well plate format using a compound library of 967 small molecules including FDA approved drugs, biologically active compounds, and small molecule kinase inhibitors synthesized in house^14,15^. MDA-MB-231 cells plated in a 96 well plate were first treated with each library compound and the relative expression level of miR-21 compared to GAPDH mRNA was evaluated after applying the PANGO sensor to the cells. The high content analysis of fluorescence signals corresponding to the Cy3-PNA-21 and Cy5-PNA-GDH inside cells in each well and the number of viable cells per well were carried out by using an IN Cell Analyzer system, a cellular and subcellular imaging system for automated imaging in live cells (Supplementary Fig. S3a).

**Figure 2:**
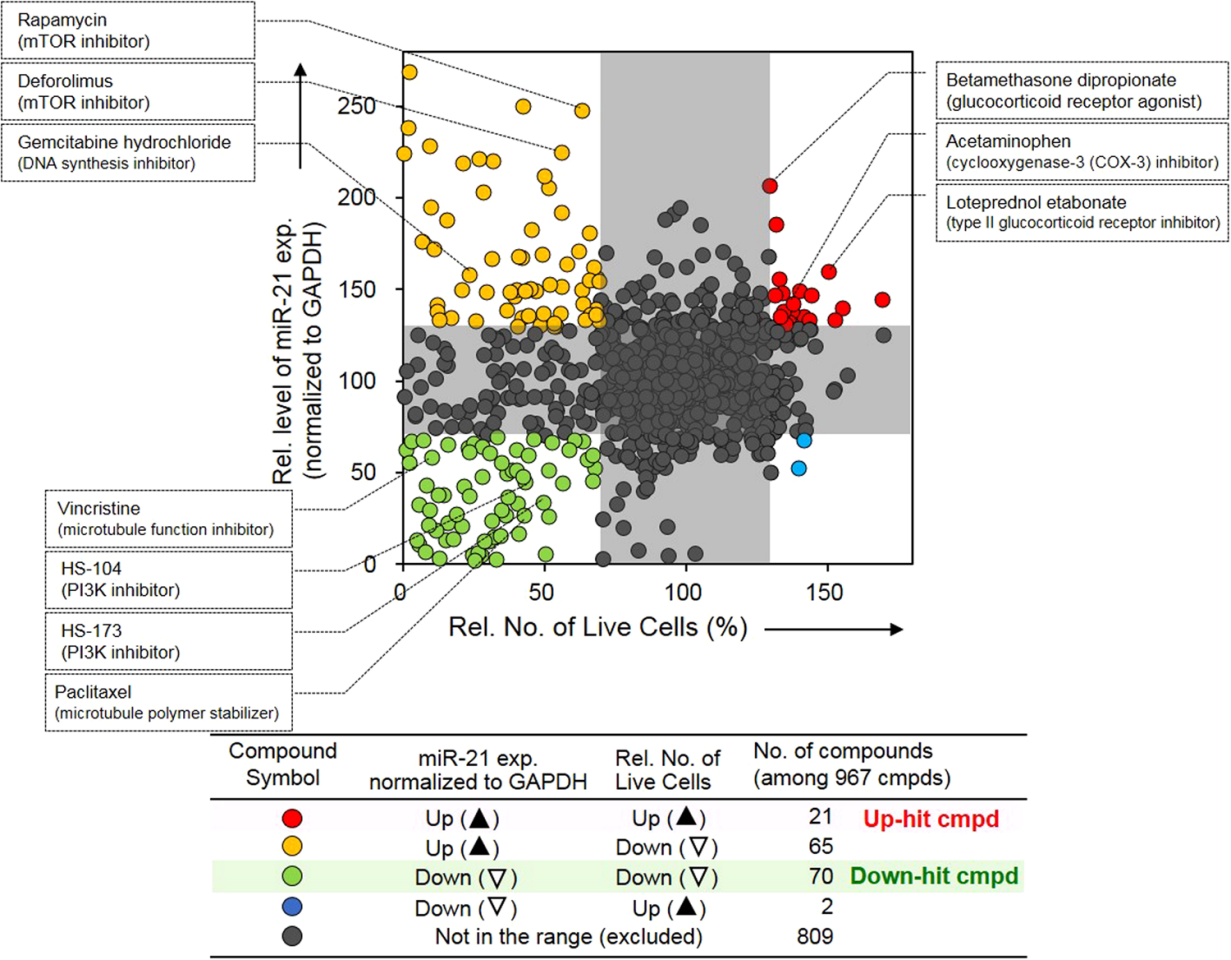
Compound screening that affects the miRNA expression change and live cell numbers. All tested chemicals were categorized into 5 groups according to miRNA expression level and the number of live cells (up: over 30%, down: over 30%) on each sample and displayed in the XY scatter plot. The screen of 967 chemicals identified 158 candidate compounds that showed the change of miR-21 expression and the number of live cells in each sample respectively after drug treatment compared with control sample. Among them, compounds that affect the up or down-regulation of both miR-21 and the number of live cells at the same time were assigned as follows; up-hit and down-hit compound, each. We found that some drugs damaging DNA belonged to the yellow-colored region (miR-21 up-regulation and cell number down-regulation), steroid anti-inflammatory drugs to the red-colored region (up-regulation of both miR-21 and cell number) and the small substances influencing the function of microtubules was fallen into the green-colored area (down-regulation of both miR-21 and cell number).

The screening result is shown as a dot plot of the relative expression level of miR-21 normalized to GAPDH expression versus a number of live cells per well in Fig. 2. To select hit compounds, changes of 30% increase or decrease relative to controls were considered as significant changes in miR-21 expression and cell proliferation after library compound treatment (Supplementary Fig. S3b). The screening of 967 compounds identified four different classes of hit small molecules consisting of i) 70 compounds that reduce both miR-21 expression and cell proliferation (green dots, referred to as “down-hit” compound), ii) 65 compounds that enhance miR-21 expression and reduce cell proliferation (yellow dots), iii) 2 compounds that suppress miR-21 expression and increase cell proliferation (blue dots), and iv) 21 compounds that enhance both miR-21 expression and cell proliferation (red dots, referred to as “up-hit” compound) (Supplementary Table S1). Analysis of the selected hits was performed to find whether there are similarities in the functions of small molecules categorized into the same group. We found that several anti-inflammatory drugs such as acetaminophen (non-steroidal)^16^, loteprednol etabonate and betamethasone dipropionate (steroidal) are among “up-hit” compounds shown as red dots in Fig. 2. Steroid hormones such as estrogen and progesterone are also found in the “up-hit” compounds. The compounds marked as yellow dots include some genotoxic drugs such as gemcitabine hydrochloride (DNA synthesis inhibition) and irinotecan (topoisomerase I inhibitor) and some mTOR inhibitors such as rapamycin and deforolimus. In addition, phosphoinositide 3-kinase (PI3K) inhibitors (e.g. GDC0941, BEZ235), histone deacetylase (HDAC) inhibitors (e.g. belinostat, SB939) and mitogen-activated protein kinase kinase (known as MAP2K or MEK) inhibitors (e.g. U0126, PD318088) were also found in the same hit group. The genotoxic drugs are often used as chemotherapeutic agents to inhibit synthesis and function of nucleic acids to treat cancers. mTORC1 is known as a target sensitive to rapamycin and deforolimus that control protein synthesis and regulate intracellular signals from various growth factors^17^. The chemicals inhibiting microtubule formation and kinase activity are among “down-hit” compounds shown as green dots in Fig. 2. Among the down-hit compounds, paclitaxel—a microtubule-stabilizing agent that induces defects in mitotic spindle assembly and cell division—is already known to down-regulate miR-21 expression in human glioblastoma cells independent on the status of phosphatase and tensin homolog (PTEN), a target gene of miR-21, in a previous report^18^. In addition, some kinase inhibitors were found in down-hit compounds that suppress both miR-21 expression and cell viability. All PI3K inhibitors used in this study decreased cell viability by blocking of Akt (also called as protein kinase B, PKB) activation inducing apoptosis inhibition^19^, and some PI3K inhibitors decreased miR-21 level as well. Finally, only two small molecules were found to suppress miR-21 expression and enhance cell proliferation at the same time but the deviation from borderline was not significant (blue dots in Fig. 2).

Prior to further investigation of the hit compounds, we performed a quantitative comparison between intensities of PANGO fluorescence corresponding to miR-21 expression and real-time PCR (qRT-PCR) data on miR-21 expression by using 3 up-hit and 5 down-hit compounds to verify the reliability of the present biosensor- based screening method (Supplementary Fig. S4). The results with PANGO sensor exhibited a good correlation with the miR-21 expression levels estimated by using qRT-PCR, giving the coefficient of correlation, R = 0.822^20^. We demonstrated that there was fairly good accordance and overall tendency between PANGO and PCR analysis, whereas there was some that fell outside the overall pattern of the relationship. According to the comparative studies of different miRNA analytical platforms, the repeatability of intra-platform was verified with the correlation coefficient values of ranging above 0.9^20,21^. On the other hand, the degree of agreement of inter-platform comparison studies showed the low accordance level around 0.5~0.7 presumably due to the lack of adequate data normalization method and some divergence in stringency of detection criteria between platforms. In several types of platforms to characterize miRNA expression level and profiling, there have been reported various factors that affect miRNA expression analysis e.g. sample preservation (e.g. frozen, formalin-fixed), and extraction methods which may cause damage in miRNA stability^22^. Thus, this result demonstrated that PANGO sensor can be potential platform showing quite efficient and well-correspondence for miRNA expression analysis along with PCR method.

### Cell growth inhibition of the selected compounds

**Figure 3:**
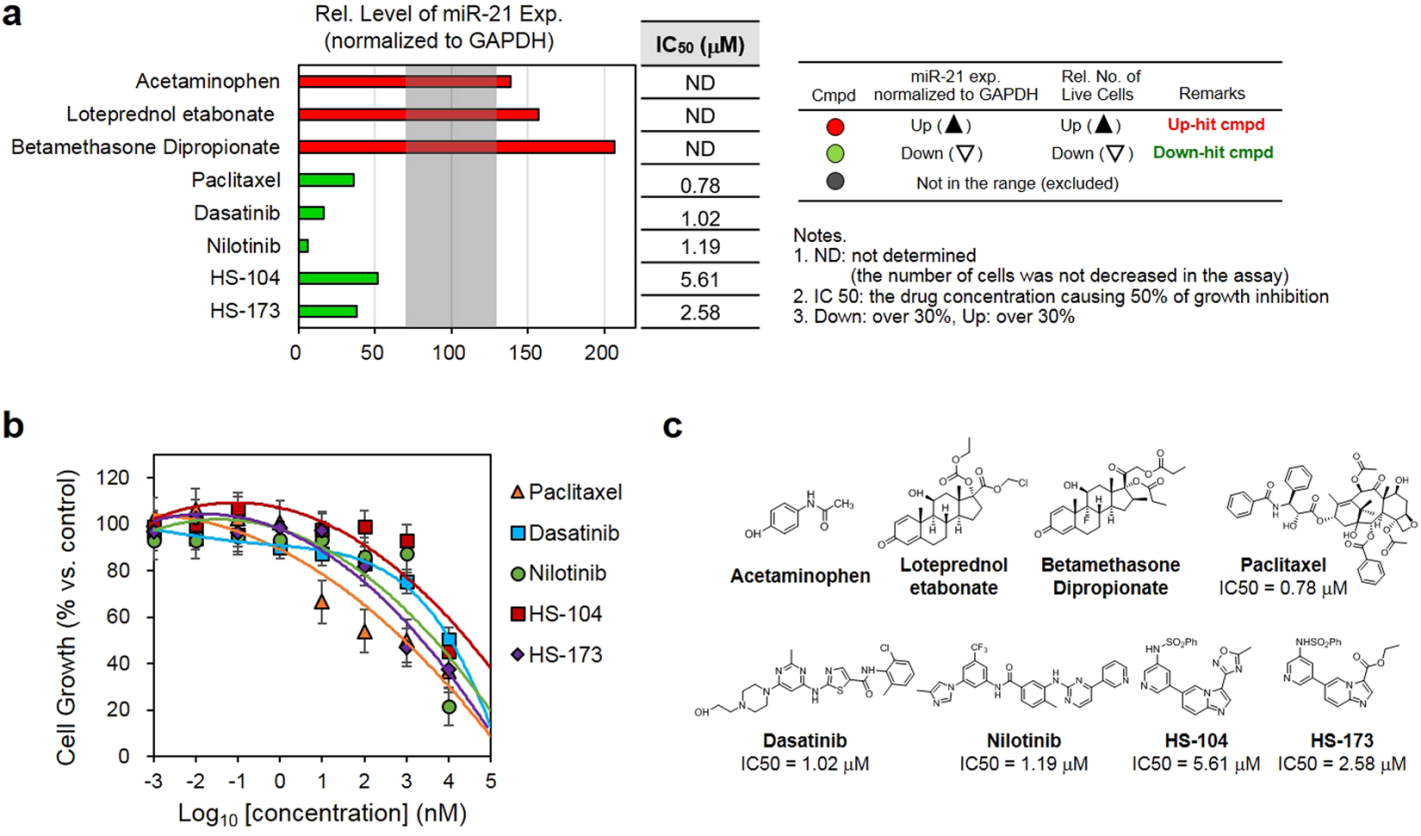
Evaluation of the cell growth inhibition effect of up- and down-hit compounds. (**a**) Chemical structures of 8 compounds selected from the compound library. (**b**) Cell growth inhibition curves obtained from the down-hit compounds. Metabolic viability was measured by using MTT assay. (**c**) The changes in miR-21 expression and IC_50_ values for 8 up- and down-hit compounds (3 up-hit compounds and 5 down-hit compounds). Data are expressed as mean ± s.e.m.values performed in triplicate.

For quantitative cell metabolic viability assay, we selected 3 ‘up-hit’ compounds and 5 ‘down-hit’ compounds of which chemical structures are shown in Fig. 3a. After the treatment of the compounds to MDA-MB-231 cells at various concentrations, we performed MTT metabolic viability assay and estimated the 50% cell growth inhibitory concentration (IC_50_). The results indicated that the down-hit compounds inhibited cell growth in a concentration-dependent manner with IC_50_ values ranging from 0.78 to 5.61 μM (Fig. 3b and c). Cells treated with the up-hit compounds did not show any decrease in cell viability in a given concentration range (0~10 μM) as expected. For further analysis of the hit compounds, we more focused on the down-hit compounds in the present study because inhibitors of miR-21 expression are often considered as therapeutically useful chemicals to treat cancers. However, we expect that the hits other than down-hit compounds will provide valuable information on the miR-21 functions with further investigation in the near future.

### Validation of cellular effects of the hit compounds on the miRNA expression change

We investigated the changes in the miR-21 target gene expression and phenotypes of MDA-MB-231 cells treated with two novel down-hit compounds including HS-104 and HS173 to correlate the miR-21 down-modulation induced by the down-hit compounds with the related protein expression level and with the associated cellular phenotype changes.

**Figure 4:**
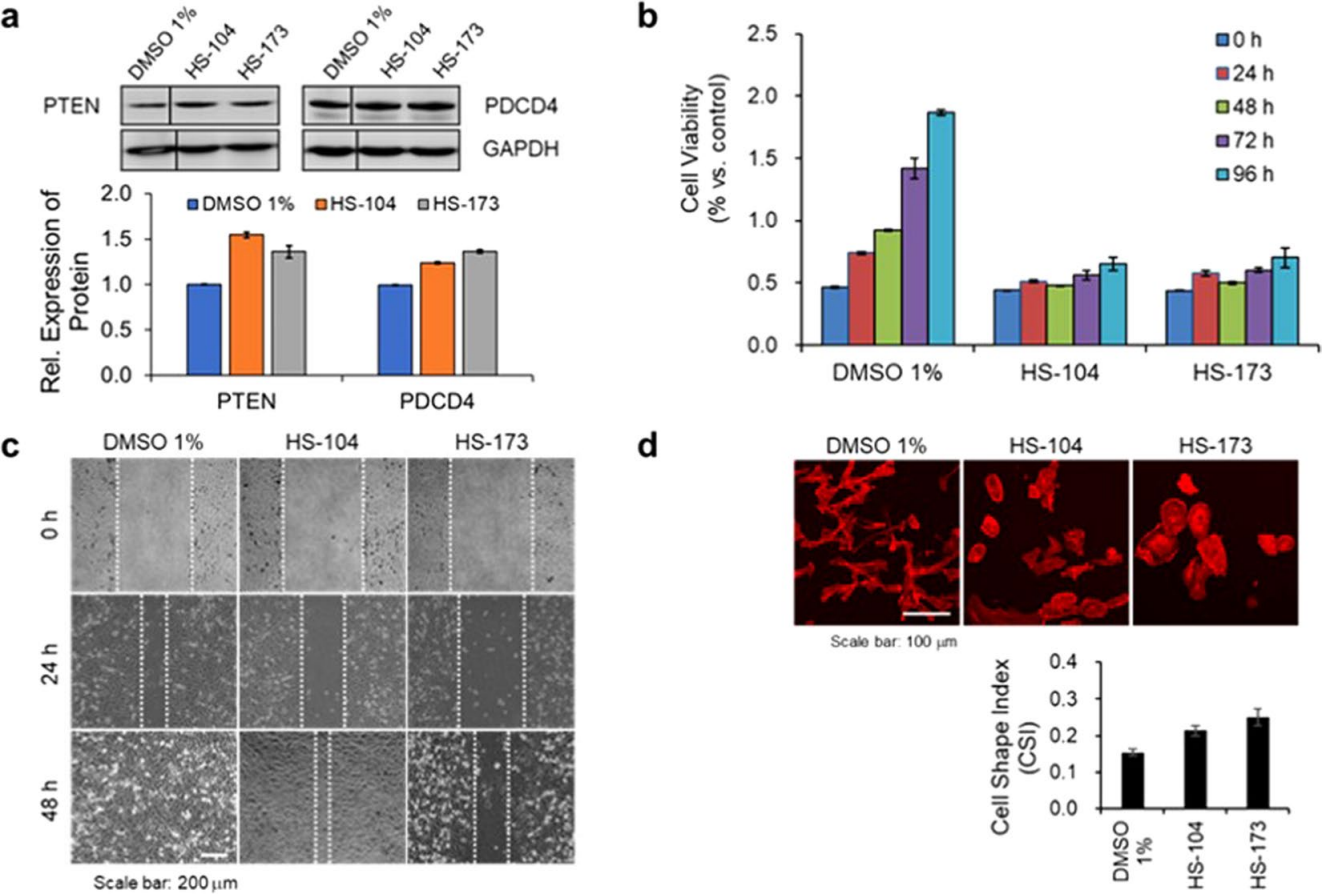
Analysis of the functional aspects of miR-21 expression changes at molecular and cellular levels. (**a**) The PTEN and PDCD4 protein level were assessed after 48 hours of compound treatment by western blotting. The GAPDH level was measured as a reference gene. The two hit compounds (HS-104, HS-173) up-regulated the miR-21 target gene (PTEN and PDCD4) expression in MDA-MB-231 cells. The blots for the same protein are cropped from the same gel and full-length blots are shown in Supplementary Fig. S5. (**b**) Cells were incubated with the PI3K inhibitors for different time periods and the metabolic viability was determined by MTT assay. (**c**) Cell migration was evaluated using a scratch wound healing assay after creating uniform scratch in cell monolayer. The retarded rate of motility was observed in cells with the two hit molecules (HS-104, HS-173). (**d**) Morphological changes of cells were observed by staining actin cytoskeleton. The cells treated with two hit compounds, HS-104 and HS-173, showed less invasive phenotypes compared to control cells, showing rounder cell morphology. Data are expressed as mean ± s.e.m.values (error bars) performed in triplicate.

To observe the target gene expression followed by the change of miR-21 expression level, we evaluated the expression of two direct targets of miR-21, PTEN and programmed cell death 4 (PDCD4)^23,24^, in the cells treated with the down-hit compounds. Several studies reported that numerous miRNAs have effects on kinase signaling pathways^23–26^ and inversely, miRNA expression can be modulated at the level of biogenesis and expression pattern by kinases^27,28^, suggesting the possibility that the miR-21 expression level is closely related to the PI3K signaling pathways. Western blot analysis revealed over 27 and 33% increase respectively in PTEN and PDCD4 protein expression upon treatment of the PI3K inhibitors, HS-104 and HS-173, selected from the down-hit compounds in MDA-MB-231 cells (Fig. 4a). Quantitative measurement of miR-21 expression in the cells treated with HS-104 and HS-173 showed significant reduction in miR-21 expression as 54 (*p* = 0.002), 66% (*p* = 0.001) decrease, respectively (Supplementary Fig. S4) in the PANGO based analysis. Collectively, the cells treated with the selected down-hit compounds showed the reduced cell viability with the increased expression of PTEN and PDCD4 (Fig. 4b). In addition, HS-104 and 173 showed lower IC_50_ values and similar or higher miR-21 suppression effect compared to conventional PI3K inhibitors such as Wortmannin and LY294002, which implied that the selected PI3K inhibitors could be more effective drugs for suppression of miR-21 with higher efficacy at lower concentrations range. It is reported that the specific signaling (PI3K in this study) and related miRNA expression pattern (miR-21 in this study) which is adjacently placed could be altered and affected in the context of inhibitor action^29^. In the present study, we found that miR-21 expression in the cells treated with the selected PI3K kinase inhibitors was down-regulated, accompanying the reduced cell viability and low IC_50_ values.

To evaluate the changes in cell invasion and migration capability, we next performed scratch wound healing assay after treatment of the down-hit compounds. Although the changes in miRNA expression is hardly defined by certain cellular phenotypes, a number of previous studies reported that knockdown of miR-21 expression led to the reduced cell proliferation, invasion, and metastasis progress^11,30^. Consistent with the reports, we found that the repair of wound gap was delayed more than 20% in the cells treated with the down-hit compounds compared to control cells, indicating retarded cell growth and migration rate (Fig. 4c)^31^.

Lastly, to evaluate the effect of miR-21 expression on cell morphology change, we observed cell shape changes after treatment of the selected compounds to the cells, followed by staining the actin cytoskeleton using fluorescent dye conjugated phalloidin. We determined the cell elongation by calculating the cell shape index (CSI) that indicates the circularity of cells, 0 for a linear shape and 1 for a perfectly round shape^32^. The CSI close to 1 illustrating a rounder shape is often inferred as less invasive morphology^33^. The cells treated with HS-104 and HS-173 particularly exhibited the morphological changes to rounder shapes showing an increase up to 60% of the CSI in the cells treated with compound HS-173 (Fig. 4d). Overall, it seemed that the reduced cell migration with the treatment of HS-104 and 173 was accompanied by morphological changes of cells to rounder shapes.

## Discussion

We developed a robust high-throughput screening strategy that enabled quantitative measurement of target miRNA expression in living cells and demonstrated a screening to identify new compounds that affect up- or down-regulate miR-21 expression along with the quantitative evaluation of relative cell proliferation rate. The sensor named as ‘PANGO’ is composed of fluorescent dye-labeled PNA and nanomaterial NGO as a sequence-specific probe towards target miRNA and as a cellular delivery vehicle of the probe with fluorescence quenching ability, respectively.

The PANGO based screening of a chemical library composed of 967 compounds identified small molecules that down- or up-modulate miR-21 expression and increase or decrease cell proliferation at the same time. Because miR-21 is overexpressed in various cancer cells and strongly associated with cell survival and drug resistance process, down-regulation of miR-21 could be a strategy for effective treatment of cancer. The ‘down-hit’ compounds we identified in the present study have the possibility to serve as therapeutically useful chemicals to treat cancers and further, can be employed in the mechanistic study of miRNA function in basic research. In this study, we selected two PI3K inhibitors among six ‘down-hit’ compounds that decreased both miR-21 expression and cell viability for further analysis of molecular and cellular changes associated with miR-21 down-regulation such as kinase signaling and cell morphology. We found that the selected PI3K inhibitors delayed cell migration in MDA-MB-231 cells, suggesting reduced invasiveness of the cancer cells. Among them, two PI3K inhibitors changed MDA-MB-231 cell morphology to rounder shape showing increased cell shape index, implicating that the reverse EMT has proceeded together with decreased level of miR-21 expression and PTEN upregulation in the target cell^34,35^.

As has been previously reported in the literature, different cellular conditions (e.g. cell cycle progression, growth factor signaling etc.) was known to play a key role as physiological triggers to lead to changes of stability and expression level of miRNAs^36,37^. In addition to that, microRNAs can be altered in their expression pattern or function upon drug treatment^38–41^. A single drug designed for a single target may function in the desired way or fall much behind expectation due to a complex cellular network and environment or back-up system, which is maintaining the current status or returning to a previous state. In this respect, the observed change of miR-21 expression level upon the chemical treatment may be deduced as a result of an indirect interaction or a secondary effect. Nevertheless, the changes can be considered as relevant outcomes shown in response to the compound treatment. To demonstrate the possible link between miRNA expression and drug treatment, we examined a series of pertinent cellular pattern studies along with miRNA expression level analysis.

The capability of multiplexed RNA sensing of PANGO enabled incorporation of internal control, GAPDH in the present study, which can be expanded to a multiplexed screening of small molecule modulators of two or more miRNA expression. We expect that the present PANGO method for miRNA modulator screening will be a great addition to the existing methods for miRNA sensing in cells and become a powerful and versatile platform technology to screen miRNA modulators for basic research related to miRNA biology and for a new class of drug discovery to treat serious diseases related to miRNA expression.

## Methods

### Preparation of nano-sized graphene oxide (NGO)

Graphene oxide (GO) sheets were synthesized from natural graphite nanofiber as starting material according to a modified Hummers’ method. After oxidation using H_2_SO_4_ (5 ml), K_2_S_2_O_8_ (0.15 g), and P_2_O_5_ (0.15 g) at 80 °C for 4.5 h, the obtained GO was cooled, rinsed with distilled water (100 ml) and dried. The dried GO powder was mixed with concentrated H_2_SO_4_ (25 ml) and then, chilled to 0 °C in an ice bath. KMnO_4_ (1 g) was added to the mixture and the temperature of the mixed solution was kept below 10 °C. After the reaction, the resulting solution was maintained in a 35 °C water bath for 12 hours and transferred into an Erlenmeyer flask (500 ml) in an ice bath. The resulting solution was stirred while slowly adding the distilled water (100 ml) into the flask at the temperature below 55 °C. Then, 30% H_2_O_2_ (5 ml) solution was added to the mixture. The solution was collected using centrifugation and rinsed with 3.4% HCl solution and acetone to remove the remained salt and acid. The obtained GO was dried under vacuum. An aqueous solution of GO was prepared by re-dispersing GO powder in distilled water.

### Small molecule compound library

A compound library of 967 molecules was prepared by combining four different sets of chemicals: 696 compounds from Bioactive Compound Library and 262 compounds from FDA-approved Drug Library (Cat. No. L1700 and L1300, Selleck Chemicals LLC, USA), 6 kinase inhibitors synthesized in house and 3 chemicals (disperse orange 3, Wortmannin and LY294002 (described as DO3, WM, and LY, respectively)).

### Small molecule screening based on PANGO

MDA-MB-231 cells were cultured in 96-well culture plates having black-wall and clear flat bottom at the density of 1.4 × 10^4^ cells per well. Chemicals from small molecule library were treated to each well at final concentration of 5 μM for 48 hours. Then, the PANGO sensor for miR-21 and GAPDH detection was added to each well in serum-free media and incubated for 12 hours. Before fluorescence signal observation, the sensor solutions were removed and cells were washed with serum containing media 5 times, followed by nuclear staining using Hoechst dye for 5 min. After addition of fresh culture media into cells, fluorescent images of cells were automatically obtained by using a high-throughput cell imaging instrument, In Cell Analyzer 2000 (GE Healthcare, UK). Fluorescence signals of two probes were acquired using excitation filter at 555 nm for Cy3-PNA 21 (exposure time: 0.9 s) and 645 nm (exposure time: 1.7 s) for Cy5-PNA GDH, respectively. The fluorescence signal of Hoechst dye was obtained using an excitation filter at 350 nm with the exposure time of 0.3 s. The images were analyzed with In Cell Analyzer 1000 Workstation software using the Multi-Target Analysis Module.

### Cell culture

Human breast cancer cell line, MDA-MB-231 cells were cultured in Dulbecco’s Modified Eagle’s Medium (DMEM) containing 4.5 g/L D-glucose and supplemented with 10% FBS (fetal bovine serum), 100 units/ml penicillin and 100 μg/ml streptomycin at 37 °C at 5% CO_2_.

### Preparation of PANGO sensor

NGO solution (50 μg/ml in distilled water) was mixed with each fluorescent dye-labeled PNA probe (10 μM per probe) in buffer (Tris-HCl, pH 7.5) for 10 min at room temperature. Optimized amount of each probe that can be adsorbed on the 1 μg of NGO is obtained by measuring the fluorescence quenching capability of the NGO, showing more than 99% of fluorescence quenching (40 pmol of Cy3-PNA 21 and 50 pmol of Cy5-PNA GDH per 1 μg of NGO, data not shown). For the detection of target fluorescence signal (Cy3-PNA 21 and Cy5-PNA GDH), sensor mixture was treated to MDA-MB-231 cells in serum-free condition for 12 hours.

### Z′-factor

The PANGO sensor solution was prepared by mixing each probe (miR-21 and GAPDH for positive controls, and probe with scrambled sequence PNA for negative control) and NGO solution in buffered solutions (Tris-HCl, pH 7.5). After 10 min incubation, each sensor solution was added to MDA-MB-231 cells in serum-free media for 12 hours. The fluorescence signals were acquired (ex/em = 550/570 nm for Cy3 and ex/em = 650/670 nm for Cy5) (n = 25) and analyzed by using In Cell Analyzer 1000 Workstation software. The Z′-factor was calculated using following equation (Zhang, J.H., *et al. J. Biomol. Screen*. **4**, 67–73 (1999)).

$${\rm{Z}}^{\prime} \mbox{--}{\rm{factor}}=1-\frac{(3{\sigma }_{{c}^{+}}+3{\sigma }_{{c}^{-}})}{|{\mu }_{{c}^{+}}-{\mu }_{{c}^{-}}|}$$

### MTT metabolic viability assay to evaluate growth inhibition

Cells were plated in 96-well plates (7 × 10^3^ cells per well) and treated with small molecules (acetaminophen, loteprednol etabonate and betamethasone dipropionate, paclitaxel, dasatinib, nilotinib, HS-104, HS-173) at a final concentration of 5 μM for 48 hours. 20 μl MTT (3-(4,5-dimethylthiazol-2-yl)-2,5-diphenyltetrazolium bromide) stock solution (5 mg/ml) was added to each sample and incubated for 3 hours to develop purple-colored formazan salt. Then, DMSO was added to solubilize the insoluble formazan salt and the absorbance of each sample was measured at 560 and 670 nm. The 50% cell growth inhibitory concentrations (IC_50_) were determined using Sigma Plot 10.0 software (Systat Software Inc., CA, USA).

### MicroRNA quantitation

MDA-MB-231 cells were seeded at a density of 1.2 × 10^5^ cells per well of a 6-well cell culture plate and treated with small molecules (final concentration of 5 μM) for 2 days. Total RNA including miRNAs was isolated using the miRNeasy Mini Kit (Qiagen, Netherlands) according to the manufacturer’s instruction. The TaqMan MicroRNA Reverse Transcription Kit (Life Technologies, CA, USA) was used for cDNA synthesis. For qRT-PCR, cDNAs were amplified with the TaqMan MicroRNA assay kit (Life Technologies, CA, USA), and the level of miRNA was normalized to that of U6 snRNA.

### Western blotting

After cells (1.2 × 10^5^ cells per well in a 6-well culture plate) were treated with culture media containing small molecules (HS-104, HS-173) at a final concentration of 5 μM for 48 hours, cells were collected and lysed for immunoblotting. Total cell lysates were separated on SDS-PAGE, transferred onto Immobilon-P membranes (Millipore, MA, USA). The blot was probed with antibodies to PTEN (Cell Signaling, CA, USA), PDCD4 (GeneTex Inc., Hsinchu City, Taiwan) and GAPDH (AbFrontier, Daejeon, Korea) (loading control) and visualized by enhanced chemiluminescence detection reagent (Life Technologies, CA, USA).

### Metabolic viability assay

MDA-MB-231 cells were plated at 5 × 10^3^ cells per well of a 96-well cell culture plate. Cells were treated with chemicals (HS-104, HS-173) at a final concentration of 5 μM or left untreated for 0~96 hours at 37 °C. Then, cells were incubated with 20 μl MTT stock solution (5 mg/ml) in each well to detect metabolically active cells. After the purple color development for 3 hours, the culture media were discarded and DMSO (200 μl) was added to each well to dissolve the insoluble formazan salt. After the absorbance reading at 560 and 670 nm (a reference wavelength), the final optical densities of each sample were taken by subtracting the reading at 670 nm from 560 nm. Cell viability of each experiment was analyzed from triplicates (n = 3).

### Scratch wound healing assay

For wound healing assay, cells were seeded in a 6-well culture plate (1.5 × 10^5^ cells per well in complete growth media). The wounds were made across the cell monolayer after the cells became confluent monolayer. To remove the floating cells, the cells were rinsed with the culture medium two times and replaced by fresh culture media containing small molecules (HS-104, HS-173) at a final concentration of 5 μM and incubated for 48 hours. For wound closure evaluation, scratched cells were observed under an inverted microscope using a 40X magnification to measure the remaining scratch widths. The horizontal distance of the migrating cells from the initial wound was measured and the measurements were analyzed by using ImageJ software available from the National Institute of Health website at http://imagej.nih.gov/ij/download/.

### Actin staining

After cells (1.4 × 10^4^ cells per well in a 96-well culture plate) were incubated with fresh culture media containing small molecules (HS-104, HS-173) at a final concentration of 5 μM for 48 hours, cells were washed with PBS and fixed with 4% paraformaldehyde in PBS (pH 7.4) for 20 min at room temperature. After permeabilization step with 0.1% Triton X-100 in PBS for 5 min, cells were stained with TRITC-conjugated phalloidin (Molecular Probes, OR, USA). Cells were imaged using an IN Cell Analyzer 2000 (GE Healthcare, UK). The cell shape index (CSI) of each cellular contour was calculated based on the ratio of cell width to cell length using NIH ImageJ.

### Densitometric and statistical analysis

The band intensities of western blots were analyzed by using NIH ImageJ (http://rsb.info.nih.gov/ij/). Data (mean ± s.e.m.) and significance (*p* < 0.05) were analyzed by using Prism (GraphPad, USA). For statistical significant testing, student’s t-test was used.

### Data Availability

All data generated or analyzed during this study are included in this published article (and its Supplementary Information files).

## Electronic supplementary material

supplementary data
