# Supplementary material for: High-throughput chemical screening to discover new modulators of microRNA expression in living cells by using graphene-based biosensor
Source: Sci Rep. 2018 Jul 30;8:11413. doi: 10.1038/s41598-018-29633-x (PMC6065314; doi:10.1038/s41598-018-29633-x)
Supplement: Supplementary file 1 — supplementary data [file 41598_2018_29633_MOESM1_ESM.pdf]

SUPPLEMENTARY DATA for

High-throughput chemical screening to discover new modulators of microRNA expression in living cells by using graphene-based biosensor

Soo-Ryoon Ryoo[1,2†], Yeajee Yim[1,2†], Young-Kwan Kim[3], Il-Soo Park[1], Hee-Kyung Na[4], Jieon Lee[5], Hongje Jang[6], Sungwoo Hong[7,8], Sung-Yon Kim[1], Noo Li Jeon[9], Joon Myong Song[10], Cheolhee Won[11] & Dal-Hee Min[1,2,11]*

[1] Department of Chemistry, Seoul National University, Seoul, 08826, Republic of Korea
[2] Center for RNA Research, Institute for Basic Sciences (IBS), Seoul National University, Seoul, 08826, Republic of Korea
[3] Carbon Composite Materials Research Center, Korea Institute of Science and Technology (KIST), Jeonbuk, 55324, Republic of Korea
[4] Center for Nano-Bio Measurement, Korea Research Institute of Standards and Science (KRISS), Daejeon, 34113, Republic of Korea
[5] Predictive Toxicology Department, Korea Institute of Toxicology (KIT), Daejeon, 34114, Republic of Korea
[6] Department of Chemistry, Kwangwoon University, 20 Gwangwoon-ro, Nowon-gu, Seoul, 01897, Republic of Korea
[7] Department of Chemistry, Korea Advanced Institute of Science and Technology (KAIST), Daejeon 34141, Republic of Korea
[8] Center for Catalytic Hydrocarbon Functionalizations, Institute for Basic Science (IBS), Daejeon 34141, Republic of Korea
[9] Department of Mechanical and Aerospace Engineering, Seoul National University, Seoul 08826, South Korea
[10] College of Pharmacy, Seoul National University, Seoul 08826, Republic of Korea
[11] Institute of Biotherapeutics Convergence Technology, Lemonex Inc., Seoul, 08826, Republic of Korea

Correspondence and requests for materials should be addressed to D.-H. M. (email: dalheemin@snu.ac.kr)

† These authors contributed equally.

Supplementary Figures

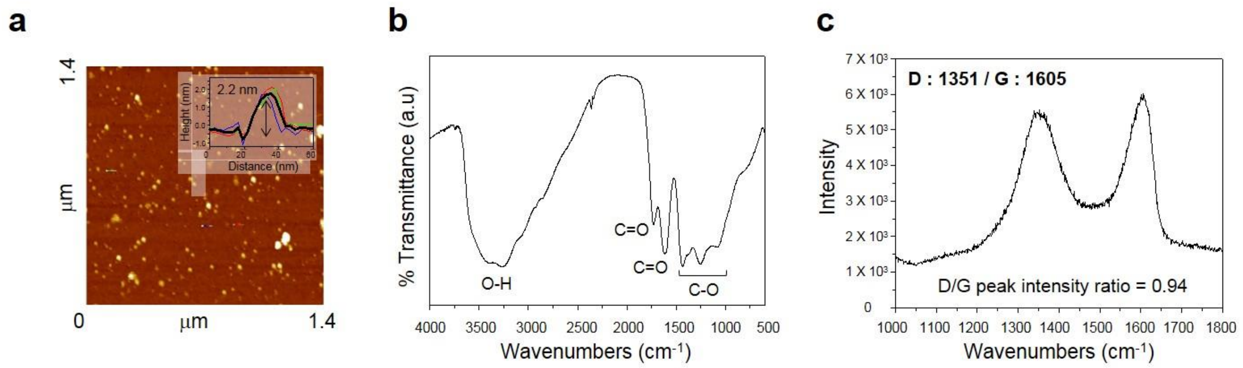

Supplementary Figure S1. Characterization of NGO. (**a**) AFM image and height profile (inset) of NGO showed the ~50 nm of diameter of NGO with average thickness 2.2 nm. (**b**) The peaks at 3398, 1724, 1595, 1429, 1261 and 1018 cm^{-1} were assigned corresponding to oxygen containing functional groups of NGO in the FT-IR spectrum. (**c**) Raman spectrum of NGO revealed peaks at 1351 and 1605 cm^{-1} which corresponded to the D- and G-band indicating the structural disordered and ordered sp^2 carbon domain, respectively.

a

Target	probe sequence labeled with fluorescence (abbreviation in the present study)	Organism
miR-21	Cy3-OO-TCAACATCAGTCTGATAAGCTA (PNA-21)	Human
GAPDH	Cy5-OO-GAGTCCTTCCACGATACCA (PNA-GDH)	Human
miR-sc	Cy3/ Cy5-ATCGAATAGTCTGACTACAACT (scPNA)	

* miR-sc was used as a negative control in this study

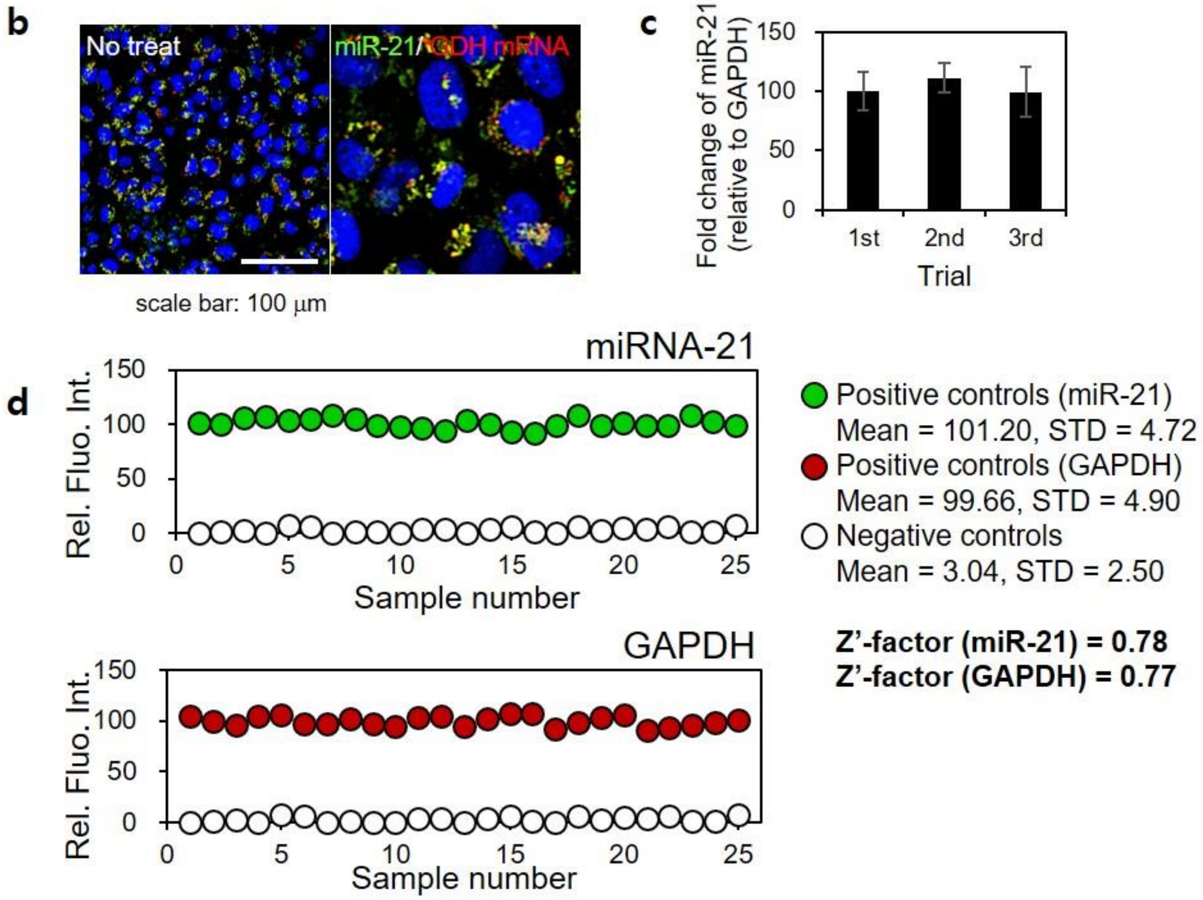

Supplementary Figure S2. (**a**) Sequences of PNA probes to detect target miRNA (miR-21) and GAPDH (glyceraldehyde-3-phosphate dehydrogenase) as an endogenous internal control. As a control, scrambled sequence of PNA (scPNA) was used and complementary miRNA towards scPNA was denoted as miR-sc. (**b**), (**c**) Fluorescence images of miR-21 (green, Cy3-labeled) and GAPDH mRNA (red, Cy5-labeled) probes from live cells without compound treatment. Fluorescence signal of each probe was observed in live cells, showing consistent fluorescent signal normalization for each sample in three independent trials. (**d**) The Z'-factor was calculated for PANGO miRNA sensor from 25 replicates of each assay. STD=standard deviation.

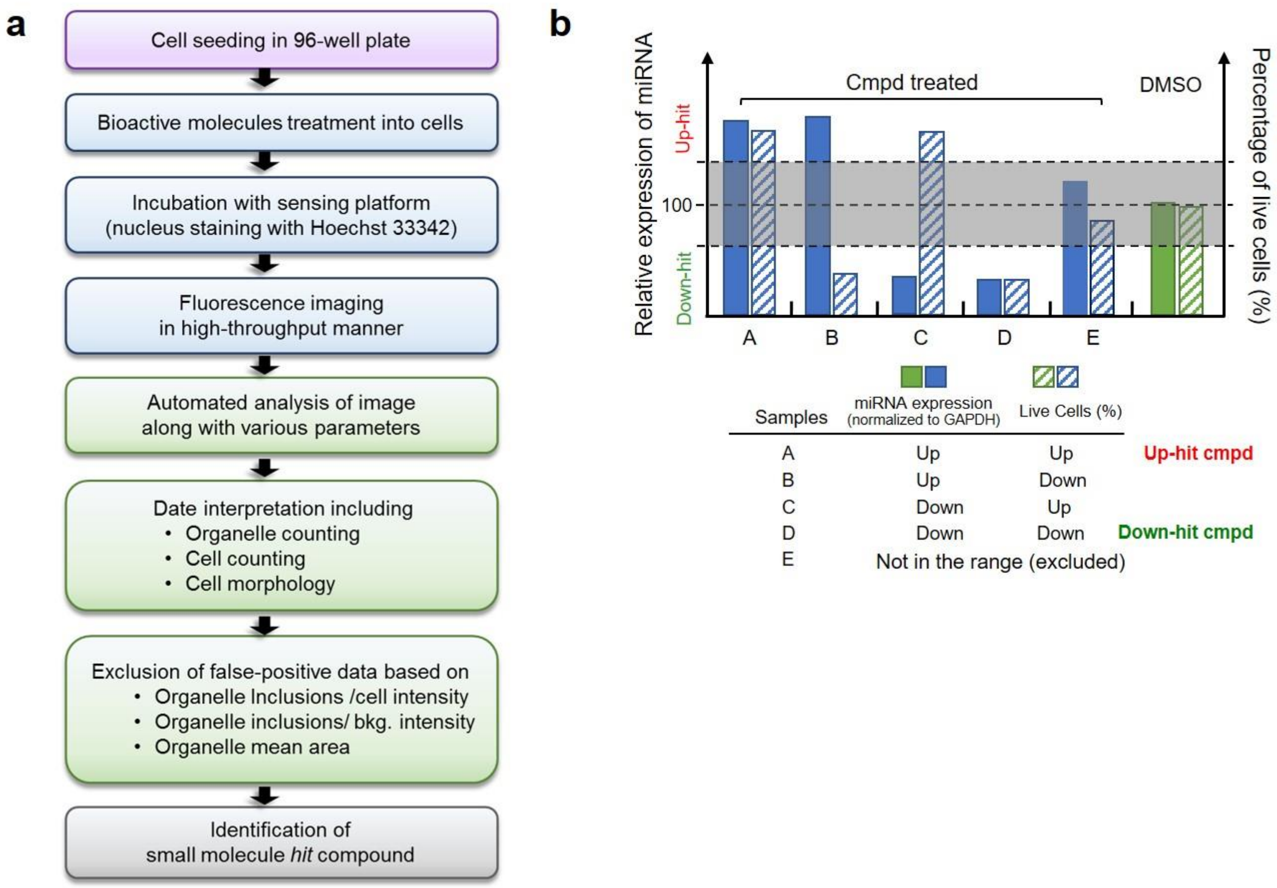

Supplementary Figure S3. (**a**) Flow chart of image-based screening process for the identification of small molecules using automated imaging instrumentation and image analysis. (**b**) The small molecule selection guide for selecting up- and down-hit compounds from chemical library (up: over 30 %, down: over 30%) based on the miR-21 expression and the relative number of live cells.

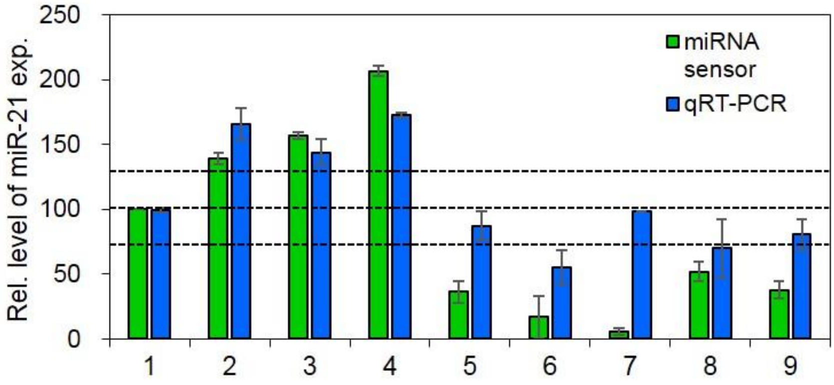

Sample	Name
1	no treat
2	Acetaminophen
3	Loteprednol etabonate
4	Betamethasone Dipropionate
5	Paclitaxel
6	Dasatinib
7	Nilotinib
8	HS-104
9	HS-173

Supplementary Figure S4. The fluorescence signal of probe and the number of cells were analyzed after test compound treatment using the PANGO and compared with the results of real-time quantitative RT-PCR in MDA-MB-231 breast cancer cells after treatment of the chosen compounds from chemical library. The PCR result exhibited good correlation with the fluorescence signal of PANGO. The data represent the average (mean±s.e.m.values) of three independent experiments.

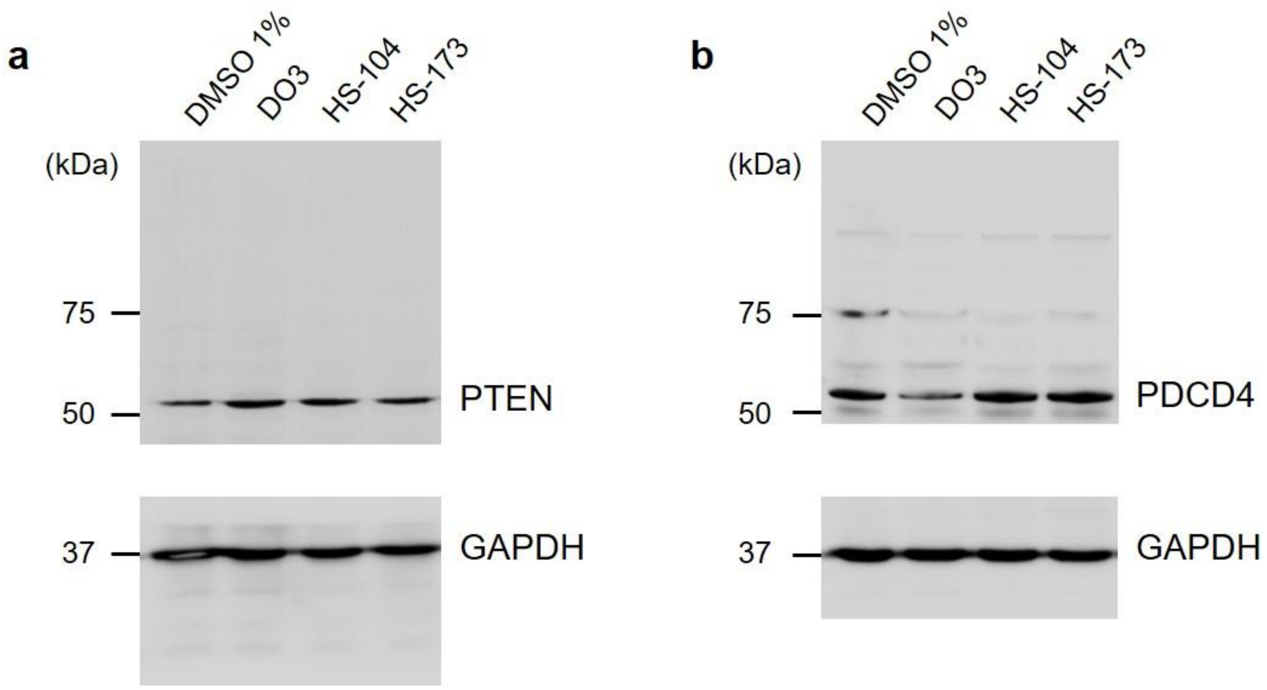

Supplementary Figure S5. Full-length immunoblot images in **Figure 4a** for (**a**) PTEN and (**b**) PDCD4. GAPDH was used as a loading control for normalization. DO3 (disperse orange 3) was used as a positive control that down-regulates miR-21 expression by inhibiting pri-miR-21 formation.

Supplementary Table

Name	Description (function, target etc.)	Remarks
Orlistat	lipase inhibitor	up-hit compound
Allopurinol	xanthine oxidase inhibitor	
Acetaminophen (Paracetamol)	Cyclooxygenase-3 (COX-3) inhibitor	
Erythromycin	macrolide antibiotic	
Ketoprofen	COX inhibitor	
Estrone	Estrogen/progestogen Receptor	
Loteprednol Etabonate	anti-inflammatory corticosteroid	
Methyltestosterone	A synthetic anabolic steroid	
Ipratropium bromide	muscarinic antagonist	
Sulfanilamide	bacterial enzyme dihydropteroate synthetase inhibitor	
Betamethasone dipropionate	glucocorticoid steroid	
Betapar(Meprednisone)	glucocorticoid	
Hydrocortisone(Cortisol)|	steroid hormone or glucocorticoid	
Divalproex sodium	autophagy	
Emtricitabine	Reverse Transcriptase inhibitor	
Progesterone	C-21 steroid hormone	
Levonorgestrel	synthetic progestogen	
Ranitidine Hydrochloride	histamine H2-receptor antagonist	
Acadesine	adenosine regulating agent	
Clofibrate	a fibric acid derivative	
Daidzein	belongs to the group of isoflavones	
ABT-888	PARP inhibitor	yellow-colored
BEZ235	PI3K and mTOR inhibitor	
Dasatinib	dual SRC/ABL inhibitor	
Deforolimus(MK-8669)	mTOR inhibitor	
Motesanib Diphosphate	inhibitor of VEGFR1/2/3, PDGFR, c-Kit and Ret	
PD0325901	MEK inhibitor	
PI-103	Autophagy	
Rapamycin(Sirolimus)	mTOR inhibitor	
Sunitinib Malate	FLT3, PDGFRs, VEGFRs, and Kit kinase inhibitor	
Temsirolimus	mTOR inhibitor	
VX-680	pan-Aurora kinase inhibitor	
BMS-599626	EGFR inhibitor	
Obatoclax Mesylate	bcl-2 Inhibitor	
AZD2281(Olaparib)	PARP inhibitor	
GDC-0941	PI3K inhibitor	
SU11274(PKI-SU11274)	c-Met inhibitor	
Belinostat(PXD101)	HDAC inhibitor	
JNJ-26481585 (Quisinostat)	HDAC inhibitor	
AG-014699	PARP inhibitor	
U0126-EtOH	MEK1,2 inhibitor	
PHA-739358(Danusertib)	inhibitor for Aurora A/B/C, Bcr-Abl, c-RET and FGFR	
BI 2536	Polo-like kinases 1 (Plk1) inhibitor	
PD0332991	CDK inhibitor	
Everolimus(RAD001)	mTOR inhibitor	

Pemetrexed disodium	antifolate and antimetabolite for TS, DHFR,GARFT	
Gemcitabine Hydrochloride	Autophagy (DNA synthesis inhibitor)	
SNS-314 Mesylate	Aurora Kinase (A,B,C) inhibitor	
WZ3146	EGFR inhibitor	
CYC116	Inhibitor of Aurora A/B and VEGF	
ENMD-2076	Aurora kinse inhibitor	
PIK-90	PI3K inhibitor	
Cladribine	adenosine deaminase inhibitor	
Decitabine	DNA Methyltransferase inhibitor	
Bleomycin sulfate	glycopeptide antibiotic and an anticancer agent	
Cerubidine (Daunorubicin)	telomerase inhibitor	
Clofarabine	inhibitor of ribonucleotide reductase and DNA polymerase	
Eloxatin	DNA/RNA Synthesis inhibition	
Etoposide(Etopophos)	topoisomerase II inhibitor	
KU-0063794	mTOR (C1, C2) inhibitor	
Fludarabine Phosphate	analogue of adenosine and deoxyadenosine	
WYE-354	mTOR inhibitor	
Adapalene	topical retinoid	
Aurora A Inhibitor I	Aurora Kinase A inibitor	
Ispinesib mesilate	Kinesin spindle protein (KSP) inhibitors	
Tipifarnib	farnesyltransferase (FTase) inhibitor	
GSK429286A	ROCK inhibitor	
AS703026	MEK inhibitor	
PHA-793887	CDK 2,5,7 inhibitor	
PIK-93	PI3K inhibitor	
AP24534	Bcr-Abl inhibitor	
NVP-BEP800	HSP90β inhibitor	
Mycophenolate mofetil	Dehydrogenase inhibitor	
SB939	HDAC inhibitor	
CCT129202	Aurora kinse inhibitor	
AT7519	multi-CDK inhibitor	
AZD7762	CHK1,2 inhibitor	
AT7867	Akt1/2/3 and S6K inhibitor	
PD318088	MEK inhibitor	
KU-60019	ATM/ATR inhibitor	
Adefovir dipivoxil	Reverse Transcriptase inhibitor	
AZD8330	MEK1/2 inhibitor	
Salinomycin	anti-steroid drug, aromatase inhibitor	
Bortezomib (Velcade)	20S proteasome inhibitor	
Erlotinib HCl	HER1/EGFR inhibitor	
Megestrol Acetate	synthetic progesteronal agent	
ABT-737	BH3 mimetic inhibitor	down-hit compound
MS-275	HDAC inhibitor	
PF-2341066	c-Met, ALK inhibitor	
LAQ824	HDAC inhibitor	
SNS-032(BMS-387032)	CDK inhibitor (CDK2, CDK7 and CDK9)	
Docetaxel	Microtubule polymer	
Paclitaxel(Taxol)	Microtubule polymer	
BIIB021	HSP90 inhibitor	

NPI-2358	vascular disrupting agent
Adriamycin	DNA topoisomerase II inhibitor
Topotecan Hydrochloride	topoisomerase I inhibitor
2-Methoxyestradiol	tubulin polymerization inhibitor
Vincristine Sulfate	microtubule polymerization inhibitor
Fluoxetine hydrochloride	antidepressant of the selective serotonin reuptake inhibitor
GSK1059615	dual inhibitor (PI3K, mTOR)
Epothilone B(EPO906)	Taxol-like microtubule-stabilizing agent
Rocuronium bromide	aminosteroid non-depolarizing neuromuscular blocker
Tenofovir	reverse transcriptase inhibitor
Repaglinide	antidiabetic drug, Potassium Channel
Risedronate sodium	bisphosphonate
Venlafaxine hcl	arylalkanolamine serotonin-norepinephrine reuptake inhibitor
Voriconazole	triazole antifungal medication
Zileuton	5-lipoxygenase inhibitor
Ziprasidone hydrochloride	antipsychotic
Zonisamide	sulfonamide anticonvulsant
Glipizide	anti-diabetic drug
Nisoldipine	calcium channel blocker
Oxandrolone	synthetic testosterone analog
Pitavastatin calcium	medication class of statins
Chlorprothixene	antipsychotic drug of the thioxanthene class
Thioguanine	purine antimetabolite
Simvastatin	HMG-CoA reductase inhibitor
Naftopidil	α1-adrenergic receptor antagonist
Fudosteine	mucoactive agent
Neratinib	HER2 and EGFR inhibitor
KW 2449	Aurora kinase inhibitor
AZD1480	JAK2 inhibitor
AG14361	PARP1 inhibitor
LY2784544	JAK2 kinase inhibitor
MLN2238	proteasome inhibitor
Mitoxantrone|	folic acid antagonist.
Bosutinib (SKI-606)	dual Src/Abl inhibitor
Dasatinib (BMS-354825)	Src, Bcr-Abl, c-Kit
Nilotinib (AMN-107)	dual SRC/ABL inhibitor
Vorinostat (SAHA)	HDAC inhibitor
Crizotinib	c-Met and ALK inhibitor
XL-184 (Cabozantinib)	VEGFR2 inhibitor
Irinotecan	Topoisomerase
Cladribine	adenosine deaminase inhibitor
Decitabine	DNA methyltransferase inhibitor
Doxorubicin (Adriamycin)	topoisomerase II inhibitor
Clofarabine	ribonucleotide reductase inhibitor
Epirubicin Hydrochloride	topoisomerase II inhibitor
Oxaliplatin (Eloxatin)	platinum antitumor agent
Idarubicin HCl	topoisomerase II inhibitor
Topotecan HCl	topoisomerase I inhibitor
Vincristine	microtubule function

Ramelteon (TAK-375)	melatonin receptor agonist	
AMG-073 HCl (Cinacalcet HCl)	treatment of hyperparathyroidism.	
Celecoxib	COX-2 inhibitor	
Adapalene	topical retinoid	
Ifosfamide	nitrogen mustard alkylating agent	
Zoledronic Acid (Zoledronate)	nitrogen-containing bisphosphonates	
Ellagic acid	antiproliferative and antioxidant	
Loratadine	antihistamine drug	
Balofloxacin	engyme DNA gyrase	
Famotidine (Pepcid)	Histamine Receptor	
HS-104	Phosphatidylinositol 3 kinase (PI3K) inhibitor	
HS-173	PI3K inhibitor	
HS-301	c-Kit inhibitor	
Paliperidone	Dopamine Receptor	blue-colored
L(+)-Rhamnose Monohydrate	naturally-occurring deoxy sugar	
Disperse Orange 3 (DO3)	transcription of the miR-21 gene into pri-miR-21	not determined
wortmannin (WM)	PI3K inhibitor	
LY294002 (LY)	PI3K inhibitor	

Supplementary Table S1. The information of screened compounds.
